# Foreign Medical and Physical Science and Literature

**Published:** 1818-11

**Authors:** 


### FOREIGN MEDICAL AND PHYSICAL SCIENCE AND LITERATURE.

*Transactions of the Physico-Medical Society of New York.* Vol. I.
(Continued from the last Number.)

ON resuming the consideration of this work, we commence with—

*A Case of Sudden Death, from a Rupture of the left Ventricle of the Heart;* by VALENTINE MOTT, M.D.

The subject of this case was a young woman, about twenty-two years of age, of a robust and plethoric habit. She had led an irregular and dissolute life, and had been addicted to the liberal use of ardent spirits. For some time previous to her death, she had made no complaint which could be considered as amounting to indisposition. Her friends recollected to have heard her make mention of slight pains, which she called *rheumatism.* They stated also, that she had entered into a marriage-contract with a gentleman, who had broken it off a day or two before her death; since which time she appeared dejected, and had been seen sobbing and in tears.

She took supper one night, as usual, and retired to bed at the ordinary time; the following morning she was found dead in bed. From the posture in which she was found lying, on the left side, with the knees drawn up, she was at first thought to be in a profound sleep. The features of her face were not distorted; nor was there any apparent contortion of the limbs.

*Dissection.*—On raising the sternum from the diaphragm, there was nothing preternatural to be seen. The lungs on each side were free from adhesions; but the pericardium was thought to be more tense than common. On opening that membrane, a large coagulum of blood presented itself, covering the heart on all sides, and completely filling the cavity of the pericardium. This, when removed, amounted to eight or ten ounces, with an ounce or two of serum.

The heart was of the usual size, and very fat. Upon lifting its apex, it was discovered that the blood proceeded from an opening in the upper part of the left ventricle, about half an inch in diameter, of an irregular lacerated appearance. The parietes of the ventricle around the opening were considerably thinner than natural; and, upon attentive examination with the fingers, a fluctuation could be distinctly discovered, to the extent of an inch on one side of the opening; and, upon pressure, flakes of a cheese-like substance were discharged.

The pericardium shewed marks of inflammation in the part opposite to the opening, to the extent of a dollar, and adhered to the heart a little above the opening. The external surface of that part of the aorta which is generally said to be within the pericardium likewise exhibited, on one side, some traces of inflammation.

The aortal, pulmonary, and ventrical valves were in a natural state.

The contents of the abdominal cavity were in a healthy state, with the exception of the liver, which seemed larger than natural, and of a pale colour. This viscus had contracted strong adhesions to the peritoneum, where it lines the parietes of the chest, abdomen, and lower surface of the diaphragm.

From this case, the author observes, may be deduced a very important fact in pathology. It teaches us that considerable disease may exist in so essential an organ as the heart, and yet be not known, or even suspected. Authors concur in opinion as to the intricacy of most of those diseases in their commencement; they are so exceedingly obscure and insidious, that the most attentive and sagacious physicians are perplexed and embarrassed. They are frequently mistaken for disease of some other part, and, in their progress, the seat of pain is often referred to various and distantly-situated organs.

We are much pleased to find that some physicians of London, whose talents admirably qualify them for the investigation, are now very sedulously engaged in researches on this interesting subject, from which we expect much important addition to our knowledge. There is, indeed, no organ of the human body the pathology of which is so little understood as that of the heart. Our ignorance in this respect may, doubtless, be attributed, in a great degree, to the imperfection of our knowledge respecting the physiology of that organ: much light may, however, be thrown on this subject by the researches of M. Lallemand on the functions of the great sympathetic nerve, which we have laid before our readers in the last and present Numbers of this Journal.

Diseases of the heart are frequent, distressing, and, sooner or later, always terminate fatally: they, therefore, urgently demand the most serious consideration from physicians, more particularly as they are likely to be still more common, as consequences of the physical and moral habits dependant on the state of civilization to which we are arriving.

*Case of Phlegmasia Dolens in a Male*; by G. B. PURDY, M.D.

Dr. Purdy was himself the subject of the disease designated by the above term. We shall give the relation of it in his own words:—

"During my attendance as house-physician in the New-York Alms'-house, in the autumn of 1814, I contracted a malignan typhous fever, which very much reduced me. Soon after the attack I perceived a swelling in the left groin, which became painful by motion; and it continued, without much alteration in size, for five or six days: after which, the fever was so severe that I had no correct knowledge of my situation until about four weeks afterwards, when I began to convalesce. I was then extremely debilitated, and laboured under all the symptoms usually subsequent to that disease. At this time my attention was again called to the tumor in the groin: it was considerably enlarged, and extremely painful on pressure, and also when the limb was extended. After remaining in this condition for several days, I was, on the 28th of September, imprudently exposed to a draught of damp cold air. My left foot soon became unusually cold, and the calf of the leg was affected with pain similar to that produced by the cramp. The pain continued to increase, notwithstanding the immediate application of warm fomentations, frictions, antispasmodics, &c.; and it soon became almost intolerable. After taking large quantities of laudanum, and pressing my foot forcibly against the bed-post, the pain was so much relieved that I fell asleep. When I awoke in the morning, my leg and thigh were swelled to twice their ordinary size; they had a shining white appearance, and the skin was smooth, tense, and elastic. The saphena major and minor veins were much distended; and the whole limb was extremely painful to the touch, or on the least motion. There was an unusual degree of heat in the part, and considerable symptomatic fever.

"The sensibility gradually diminished for about two weeks, but the swelling continued much the same. At this time, by the constant use of bandages and stimulant applications, its size was so much reduced, and its strength increased, that I was soon able to walk with the assistance of a cane. During the succeeding winter I attended the medical lectures, and, by keeping the limb constantly bandaged, and taking moderate exercise, its strength increased, and I flattered myself that it would soon regain its former vigour. On the 6th of July, 1815, I perceived that my leg was much weaker than usual, and enlarged about an inch in circumference; but by rest, friction with stimulating applications, and cold bath, it has since regained its former condition of imperfect strength, from which I fear it will never recover."

*Case of an Artificial Joint cured by Friction;* by John Meeker, M.D.

A lady, aged twenty-eight years, fell from a chair on which she was standing, and fractured the radius about four inches above the carpus; the arm was much bruised; and the tendon of the biceps flexor cubiti muscle, coming in contact with the edge of a pail on which she fell, was wounded near its insertion. The arm was dressed lightly in consequence of the bruise, and one splint applied. The swelling that followed was so considerable, and the pain so acute, that the dressings were removed, and a camphorated lotion used to the arm; the former were not steadily re-applied for three weeks, owing to the pain and inflammation which continued during that time. In about six weeks the muscle had recovered its power, and the arm was reduced to its natural size and appearance; but pronation and supination produced pain, and a sensation of something loose in the arm.

Dr. Meeker saw the lady two months after the accident, and found that the radius had been fractured, and an artificial joint formed. The attending surgeon had not detected the fracture, and, consequently, could not account for the symptoms then existing.

As she expected soon to visit Philadelphia, she wished to delay any attempt at a radical cure until she could have the advice of Dr. Physick. In the mean time, about three months after the accident, on giving the hand of the injured arm to a friend, he shook it so heartily that it produced excruciating pain, from the friction of the ends of the bone at the artificial joint. The arm remained sore, and in a few days slight lancinating pains were felt in the part, which remained for some time. At the end of three or four weeks she had regained the free and perfect use of the arm.

This case is not calculated to excite much hope of success from the same means, in the generality of the unfortunate cases of artificial joints, which appear to arise from some undistinguishable peculiarity of constitution. In the present instance, the want of union may be attributed to the neglect of the use of means to keep the ends of the bones adapted to each other immediately after the accident. Friction of the ends of the bone is the mode of practice advised by Celsus, and Mr. Hunter and many other modern surgeons; and several instances are related in which it has been successful, but it has more frequently failed. The practice proposed by Dr. Physick, of passing a seton through the joint, has not answered the expectations it once excited. It has, however, succeeded in several instances, one of which occurred to Baron Percy: an account of this case was read to the Ecole de Médecine at Paris, in 1805, by M. Laroche. This was a case of compound fracture of the thigh, which appeared to have lost all disposition to unite; in two months the patient was able to walk without crutches. Mr. Wardrop employed it in one case with some advantage, as coalition of the bones took place, but it was not firm union. It has been successful in one instance, in which it was practised by Mr. Brodie, on the broken thigh of a boy aged 13. In another case, of a man about 30 years of age, whose thigh had remained disunited a little below the middle for the space of a year; firm coalition of the bones had taken place after a few weeks; but, on the patient getting up to walk about, the seton being withdrawn, the uniting medium became gradually soft, and at length appeared to be absorbed. A long continuance of the use of the same means, subsequently to this, was ineffectual. The seton sometimes produces so great a degree of irritation as to render the removal of it necessary before the object of it has been accomplished. This occurred in a case in which it was employed by Dr. A. Copland Hutchinson, (related in his *Pract. Observ.* page 162.) Blisters, kept open for a considerable time, have been used with success in a few instances.

Dr. Meeker observes, that he presumes the practice of friction will be found more effectual in cases of preternatural joints in the fore-arm and leg, where one bone only has been fractured, than in those of the thigh; the sound one preventing the fractured ends from passing each other, which occasions absorption of the edges of the surface, and leaves them of a conical form,—thus diminishing the approximating surfaces.

### *An Account of a Family Predisposition to Hæmorrhage;* by Drs. W. and S. Buel.

This very remarkable and interesting phenomenon occurred in the family of Oliver Collins, the son of the minister of the town of Litchfield, Connecticut. It could not be ascertained that there was any indication of this disposition in any of the predecessors of the children of the person above alluded to, either on the male or female side. He had several sisters and brothers, who had children to the second and third generations, in none of whom has there been any manifestation of the hæmorrhagic trait.

Oliver Collins had four sons and two daughters. In all of the sons, this predisposition discovered itself from early infancy, and continued through life. Three of them bled to death: it is not known where the fourth is, or whether he be alive. Two of them, although frequently reduced by hæmorrhage to a state of exhaustion threatening the extinction of life, lived to adult age. One of them eventually died from hæmorrhage, induced by the extraction of a tooth; the other from hæmorrhage following a slight contusion of the fore-finger. The one which died in childhood, bled to death from a slight laceration of the tongue by the teeth, occasioned by a stroke under the chin.

The next appearance of a disposition to bleeding was in a son of Mrs. Baldwin, one of Oliver Collins's daughters. He suffered from it in an equal degree with his uncles. He lived till near the age of puberty, when he bled to death from a slight wound caused by the fall of a pewter-plate on his foot.

The last instances in which this family characteristic has discovered itself are the male children of Mrs. Kilborn, a daughter of Mrs. Baldwin. She had four sons, in all of whom a tendency to bleeding has been strongly marked since early infancy. In her daughters it has been absent. Two of the sons have already, in childhood, fallen victims to hæmorrhage. Another son, who was drowned at the age of eighteen months, had exhibited the hæmorrhagic predisposition as strongly as his brothers, and once bled almost to death from a rupture of the frænum of the upper lip. The death of one of the boys was caused by a hæmorrhage proceeding from a wound produced by a stump of wood penetrating the bottom of the foot. He died in about forty-eight hours from the time of receiving the injury. His brother died after extreme exhaustion induced by nasal bleeding. He had an abscess in the leg, which discharged a short time previous to his death, and which might have co-operated with the loss of blood in exhausting the powers of life. The only surviving son has been frequently reduced to a state of debility threatening life, by hæmorrhage from the nostrils, small lacerations of the frænum of the upper lip, and slight wounds in various parts of the body.

The other daughter of Oliver Collins has both male and female descendants, in none of whom has this family peculiarity of constitution appeared. It cannot be discovered that there has been, in any instance, in the female descendants of the Collins' family, any evidences of the hæmorrhagic trait.

It is only the instances which occurred in the children of Mrs. Kilborn which came under the observation of the Doctors Buel; the information of the fate of the other victims was derived from their relations.

It is remarked that, in the Kilborn boys, there is not only a strong disposition to hæmorrhage when the blood-vessels are wounded, but that there is also a tendency as strongly evinced to plethora and spontaneous hæmorrhage. They have all of them had frequent bleeding from the nostrils, gums, and particularly from the frænum of the upper lip.

In all instances, slight contusions were followed by extensive ecchymosis, and a great degree of tumefaction. The boys, when beginning to walk, have had for a number of months perpetual ecchymosis about the nates, from the contusions received by falling on them. The plethoric state of the system, which took place whenever any considerable time elapsed without hæmorrhage, was evinced by a flushing of the face, sense of fulness about the head, indisposition to active exertion, and all the usual symptoms of plethora. Their countenances had always a sallow, pallid hue. This sallowness was not absent when the blood-vessels had a plethoric fullness; the face was flushed, exhibiting a complexion strikingly different from the red and white tints of health.

No wounds were ever witnessed of sufficient size to have the current of blood, in case it was arterial, assume the *per saltem* motion; but the strong impetus with which the blood was discharged, and the powerful resistance it opposed to all remedies, whether mechanical or of other kinds, in whatever manner applied, would seem to be strong evidence that it was arterial. The blood was not destitute of coagulability; large coagula were frequently formed about the orifices of the wounds. When by any means the hæmorrhage was suppressed, the part above became distended, livid, and intolerably painful, and relief was obtained only by permitting a recurrence of the bleeding. Slight wounds, in some instances, healed by adhesive inflammation. It was noticed in some of Kilborn's boys, that they had, during the debility existing after hæmorrhage, depraved appetites: one of them was disposed to eat sand; another, common earth.

The authors state, that they had not discovered any mal-conformation or unnatural distribution of the blood-vessels generally, nor in the valvular construction of the veins particularly. They observe, that the foregoing facts are such as occur to their recollection: it was not until some time after witnessing them that a statement was requested from them for publication; otherwise, they might have been more attentive in their observations, and have obtained additional facts to assist the pathologist in forming a theory of the affection.

We regret exceedingly that so little is related of the appearances on dissection; nothing whatever is said respecting the heart. We witnessed the examination of the body of a young man who died from nasal hæmorrhage, and who was represented to have been subject to bleeding to a great extent from the slightest causes, in which the heart was found to be of four or five times the usual size, but without any marks of disease. The extraordinary bulk of that organ arose from a preternatural development of its muscular structure.

*(To be concluded in our next.)*

---

*Pathological Observations, illustrative of some Points in Physiology*; by F. Lallemand, M.D. &c.

*(Continued from p. 348.)*

Our notice of this work, in the last Number, terminated with the anatomical description of an acephalous fœtus;—on resuming this subject, we commence with the reflections of the author on this interesting phenomenon.

We may here observe, that the heart and other parts of the fœtus, of which no mention was made in our account of its anatomical formation, did not show any deviation from the natural state.

M. Lallemand prefaces his reflections with the following question—"To what causes were the destruction of the brain, &c. and the bifurcation of the spinous processes of the vertebræ, to be attributed?" The state of the mother during pregnancy, he observes, the general serous effusion into the cellular texture, and the dropsy of the peritoneal cavity from which she had suffered, naturally leads us to the primary cause of the disease of the fœtus, which must be attributed to the state of the blood it received from the mother. The immense quantity of water which escaped from the uterus indicates the nature of that disease, and confirms the opinion of Morgagni, which is now generally adopted. He thinks* there is not a single instance of want of brain and spinal marrow related by authors, in which the former existence of these organs could not be demonstrated; and that the cause of their destruction has been dropsy of those parts. When the disease has not proceeded so far as to rupture the integuments, it is not unusual to find, instead of brain, a considerable quantity of limpid fluid,† sometimes discoloured, and of a bloody appearance.‡ In

---

* De Sedibus et Causis Morborum, 12, 14.

† Fontanus, Ephem. cur. Nat., decur. 1, an. 3, observ. 129.

‡ Morgagni, Bonetus, Wepfer.

cases of hydrocephalus in the fœtus, the serum may be extravasated either between the dura mater and brain, or within its ventricles. The first species is very rare, and rather produces compression than destruction of the brain: the few observations we find related shew that the brain, in these instances, is reduced to a very small size, but is not destroyed:* the latter, which is by far the most common, is, as Morgagni has demonstrated, the real cause of destruction of the brain in acephalous fœtuses. M. Lallemand considers that the destruction of the spinal marrow may be attributed to the same causes. This was the opinion of Brunner† and Morgagni.‡ M. Portal mentions having seen a perfectly-formed canal in the spinal marrow, running to a great extent, in a subject that had spina bifida.§ Bauhin, Malpighi, Portal, and Gall, maintain that this cavity exists naturally; but this opinion, it would appear, has been formed from pathological observation: its existence has only been demonstrated, in ordinary cases, by making mercury pass from the ventricle of the cerebellum into the pouch by which it terminates, and then into the spinal marrow; but the softness of that nervous mass, the weight and divisibility of the mercury, must prevent our acceding to the interpretation they give of this circumstance. No instances have been witnessed of the destruction of the spinal marrow, without that of the brain. Morgagni, it is true, quotes two from Raygeri,|| in which the spinal marrow was wanting, although the brain was not entirely destroyed, but it was considerably altered in structure. These observations, therefore, instead of being in opposition to the others, serve rather to confirm them. Instances of effusion of serum into the ventricles of the brain are as frequent as those in which it takes place within the spinal marrow are rare,—a circumstance which would not happen were there a free communication between a cavity in the spinal marrow and the ventricles of the brain. The following conclusions, it appears, may be deduced from the facts above related:—1°. That a natural cavity within the spinal marrow does not exist. 2°. That the extravasation of serum into that substance arises from its having previously taken place into the ventricles of the brain. 3°. That this extravasation of fluid is the cause of the destruction of the spinal marrow. 4°. That the primary cause of this disease may frequently be attributed to the nature of the fluids communicated from the mother to the fœtus.

The bifurcation of the bones of the vertebræ, witnessed in the case of M. Lallemand, appears to be of very rare occurrence; since Ruisch, who had examined six cases, did not think it ever

---

* Vide Paré, l. 7, c. 1.—Stegman, Sepulch. l. 1. § 16, in addit. obs. 11.—Velsius.—Morgagni, lit. 12, No. 13.

† De Fœtu Monstruoso et Bicipite, dissert. in 4to.

‡ De Sed. et Causis Morb. lit. 12, No. 11.

§ Anatomie Médicale, art. *Moelle.*

|| Ephem. Nat. Cur., decur. 1, an. 3, observ. 280; et an. 8, obs. 64.

took place:* Morgagni was of the same opinion.† The two points of ossification which form the rudiments of the bodies of the vertebræ become very early united; their separation cannot be effected after the third month: the disease must, therefore, have made a considerable progress before that period, to produce the appearance above mentioned.

The passing of the œsophagus through this opening in the vertebræ; the obliteration of the canal near its insertion into the stomach; the dilatation of its superior part into a sac, filled with meconium similar to that which existed in the large intestines, prove, the author observes, more decidedly than the most satisfactory reasoning on the subject—

1°. That it is not by receiving into the stomach the fluid contained in the amnios, that the fœtus is nourished; an opinion still adopted by some men of eminent talents, notwithstanding the facts adduced of fœtuses being born without a head, and others without mouth or nostrils.

2°. That it is not the fluid of the amnios which produ cesthe meconium, because this substance was found in the usual quantity in the large intestines: if it be objected to this, that the fluid in question might have passed into the stomach from the œsophagus not being entirely obliterated,—it may be asked, why it was collected in so large a quantity in the superior part of the œsophagus? and why its accumulation had produced a dilatation of it to such an extent?

3°. That the colour of the meconium is not owing, as is generally supposed, to the presence of bile; because that which was collected in the upper part of the œsophagus was of the same colour as that in the rectum: it cannot be supposed that the bile could pass from the stomach to that situation, since there is no reason to suppose that it would overcome an obstacle which had arrested the meconium. The question then is,—in what manner is it formed? If it be not the residuum of the fluid of the amnios, it is evident it must be produced by secretion from the mucous membranes; and that this takes place in the fœtus, although it does not digest food, is supported by the fact that it continues to be formed in the inferior portion of the intestinal canal, in persons who have an artificial anus in a part of the intestines at some distance from this extremity; in which cases, the fœcal matter had not come in contact with those parts for several years. Admitting this account of its origin, it readily explains why that portion found in the mouth and œsophagus had accumulated above the constriction; why it was found, as usual, in the large intestines; and why the quantity of meconium found above the constriction, compared with that in the intestines, was in proportion to the extent of the mucous surfaces.

It is evident, from a considerable number of facts, that fœtuses have been born at, or very near, the full period, without cerebrum,

---

* Observat. Anatom. Chirurg.; observ. 34.

† De Sed. et Causis Morb.; letter 12, No. 11.

cerebellum, or spinal marrow: this is undoubtedly one of the most curious phenomena that pathology offers for the contemplation of physiologists. Before entering into the discussion of this subject, observes M. Lallemand, a question necessarily presents itself for solution—"Have these fœtuses survived the total destruction of those organs?"

M. Legallois, when speaking of this subject, says, "It would be very important to ascertain if those fœtuses were born alive or dead; but this is a circumstance which authors have not always been so attentive as to relate. I know only two in which we are assured that they were born alive.* To induce us to admit facts so extraordinary, new and well-authenticated observations are necessary."

Those cases, M. Lallemand observes, the authenticity of which is questioned by M. Legallois, are related in a manner which will not induce us to place the least confidence in them; particularly that of Mery, who does not say that he himself saw the child alive. The few words in which the description of the state of the parts is expressed, are sufficient to cause doubts respecting the destruction of the spinal marrow:—"The dura and pia mater formed a canal in the cavity of the vertebræ:" this does not accord with the observations of authors in general. The instance related by Fauvel is less extraordinary. The fœtus, which forms the subject of the observations of M. Lallemand, according to the account of the mother, gave signs of life two days before delivery. It is not impossible that of Fauvel may have shown signs of sensation for two hours, since experiments on animals asphyxiated, and the facts observed in the practice of midwifery, prove that the fœtus may live nearly as long without respiration, after communication with the mother has ceased. Now the only difference, the author observes, existing between an ordinary fœtus, and one deprived of brain and spinal marrow, which had not died before birth, is, that in the latter respiration could not be established.

Although the correctness of the account given by Fauvel may be doubted, still the opinion which M. Lallemand wishes to establish may be equally well maintained, if it be proved that fœtuses have survived the destruction of the brain and spinal marrow. All

---

* The cases to which M. Legallois refers, are related in the History of the Royal Academy of Sciences of Paris. In the volume for the year 1711, we read that "M. Fauvel presented to the Academy a fœtus without cerebrum, cerebellum, or spinal marrow; although, in other respects, well formed. It was born at the full period, lived two hours, and shewed signs of sensation when water was poured on its head during the baptismal ceremony."

In the volume for the year 1712, it is stated that M. Méry had seen a male fœtus, which was born without brain or spinal marrow, that lived twenty-one hours, and took some nourishment. The dura and the pia mater formed a canal in the vertebral cavity.

the instances related seem to show that they were alive a short time before birth. In the whole of them it is stated, that they were plump and well-formed. Morgagni particularly observes, in one instance, that no unpleasant smell arose from the fœtus ; nor was the epidermis separated in any part. In the case before us, it appears that the disease must have existed a long time before the ossification of the vertebræ, since the spinous processes were separated; and, what proves it more fully, the union by actual cicatrization of the integuments of the cranium, and the enormous pouch they formed (extending as low as the lumbar vertebræ), seem to shew that rupture of them had not taken place until they had been distended to a very large size. After the evacuation of the brain and spinal marrow, union of the integuments, it appears, had taken place: the process of cicatrization shows that inflammation had existed, and inflammation necessarily indicates life. It must then be admitted, that this fœtus continued to live until the moment of its birth, notwithstanding the want of brain and spinal marrow.

Life, in the fœtus, observes the author, is wholly employed in its development; but nutrition, in this case, being performed without the aid of respiration and digestion, is confined to phenomena which take place in each organ, but which are not cognizable by our senses. It is, however, certain that each part receives from the blood the fluids which it elaborates, and returns into it those which are no longer necessary to it;—nutrition supposes a circulation, active and regular, and consequently well-performed motions of the heart. M. Lallemand confines his reflections to the latter circumstance, from its presenting phenomena so evident as to be readily ascertainable.

It is clear that one of the two following opinions must be adopted: either that the action of the heart depends on a particular power, independent of the nervous influence; or that this nervous influence, of whatever kind, must be derived from other sources than the brain and spinal marrow. The question, thus simplified, renders the consideration of the systems of Des Cartes, Laboé, Borelli, Willis, and Boërhaave, quite unnecessary.

The doctrine of Haller on irritability is universally known, and his opinion that the action of the heart arises from the stimulus of the blood. The most important facts adduced to prove that this action is independent of nervous influence, are—that it continues to contract after decapitation and the removal of the spinal marrow, and, indeed, after its separation from the rest of the body; that mechanical irritation, electricity, and galvanism, applied to the nerves of the voluntary muscles, either during life or after apparent death, cause contraction of those muscles, but do not produce a similar effect on the heart, when applied to the cardiac nerves ; and that diseases, and experiments, which produce convulsions in the voluntary muscles, occasion no change in the action of the heart. But, in the first place, cutting off the communication of the heart with the brain does not prove any thing, if it can be shown that that organ is not the only source of nervous influence; and Legallois has demonstrated, by the most satisfactory experiments,* that those actions of the heart are not sufficient for maintaining the circulation of the blood. He compares, with much propriety, these ineffectual actions to those which are produced after apparent death in the voluntary muscles, by irritating the nerves which are distributed in them: thus the blood, the natural stimulus of the heart, will produce in that organ the same effect as other irritative causes on other muscles. He very properly establishes a difference between those actions which he considers are produced by irritability, and those which depend on nervous influence. The unaffected state of the heart during convulsions of the voluntary muscles, and the different results obtained from galvanic experiments, &c. on the nerves of the heart, and those of other muscles, prove, in the opinion of SCARPA (*Tabulæ Neurologiæ*, § 20), a difference in the nature of their nerves. It is principally on those observations and experiments, that Bichât has established his division of the nervous system of *animal life*, and the nervous system of *organic life.*

These opinions are powerfully supported by the results of the experiments made by Mr. BRODIE, to ascertain the mode in which the vegetable poisons destroy the life of animals.† The eleventh experiment, in particular, contains so many facts illustrative of this subject, that we feel disposed to transcribe the principal circumstances respecting it.

Three ounces of strong infusion of tobacco were injected into the rectum of a dog: it almost immediately produced tremulous contractions of the voluntary muscles. In five minutes the injection was repeated. The dog became sick, and threw up some of the infusion from the stomach; he became faint, and died ten minutes after the second injection. The thorax was opened immediately after respiration had ceased. The heart was extremely distended, and without any evident contraction, except slight occasional contractions of the appendix of the right auricle. Mr. Brodie divided the pericardium; but, from the great distension of the heart, this could not be done without irritating its muscular fibres with the point of the scalpel: both auricles and ventricles immediately began to contract with considerable force, so as to restore the circulation. Artificial respiration was then produced, and the circulation was kept up for more than half an hour, beyond which time the experiment was not continued.

We find, from the above facts, that the energy of the heart may be so far diminished as to be unable to propel the blood after the lungs have ceased to become dilated, and yet retain that excitability which appears to belong to muscular fibres. The cessation of respiration from the immediate influence of the poison is to be taken into consideration in this instance; but that is not

* Experiments on the Principle of Life, p. 27 *et sequent.*

† Philosophical Transactions, anno 1811.

sufficient to account for some of the phenomena,—the great distension of the heart, and the discontinuance of its action at the same time with that of respiration. Mr. Brodie's experiments shew that, when death is produced by some other poisons, as the woorara,* which act immediately on the cerebral system of nerves, the heart will continue to act vigorously for some minutes after respiration has ceased. The state of the heart in this experiment may, perhaps, be attributed both to the cessation of respiration, and the loss of part of its energy,—viz. that dependant on nervous influence. That the action of the heart, in this instance, did not cease merely from the want of respiration seems to be proved by the ninth and tenth experiments, which show that the action of the heart, in an animal poisoned by tobacco, ceases before the entire cessation of respiration. This distended state of the heart could not arise from the want of muscular irritability; for it continued to carry on the circulation with as much force, in this instance, after artificial respiration was produced, as in those instances in which decapitation of the animal only had been effected; and Mr. Brodie found, that the heart and voluntary muscles of animals killed by tobacco, were as readily stimulated to action by the influence of the voltaic battery, as if they had been killed in any other manner.

If the heart be not subject to nervous influence, observes M. Lallemand, why does it receive nerves? Since the work of Scarpa has appeared, can we say, with Soëmmering, that the cardiac nerves do not penetrate into the muscular fibres of the heart? or, with Fontana, that they have no known use? And how is it, that the heart is so particularly submitted to the influence of the passions? These objections, the importance of which did not escape Haller, produced frequent contradictions with his own opinions whenever he endeavoured to reply to them. Thus, many physiologists of his school have modified his theory of irritability, in admitting nervous influence.

If we cannot conceive the possibility of regular and powerful action of the heart, without the intervention of nervous influence; if we cannot suppose the source of this nervous power to be in the cerebrum, cerebellum, or spinal marrow,—we are naturally induced to admit, with Bichât, that the nervous system of ganglions is destined for the functions of the organs of nutrition; and that the nervous centres which compose it form a distinct system, independent of that destined for the functions of animal life. The results of the experiments of Legallois may, however, be adduced in opposition to this opinion: he observed, that the destruction of any portion, however small, of the spinal marrow, produced instant death in the parts which receive their nerves from it, and very quickly a diminution of the force of the circulation, which ceased entirely some time before the last motions of the heart. We refer our readers to those experiments for further information on this

* See Experiments 19 and 20.

point: we must, however, remark, that it cannot be admitted that the nervous system of ganglions can have continued its functions after the destruction of the spinal marrow, without concluding that these functions are entirely independent on it: it is, on the other hand, very possible to conceive that *sudden* destruction of the spinal marrow may have produced such a degree of disturbance in those functions as to cause a cessation of the action of the heart, without being obliged to conclude that the great sympathetic nerve has its origin from the spinal marrow, and that it thence derives all its power. Legallois observes, in another place, that, by proceeding gently, he could separate a considerable portion of the spinal marrow, without producing death.

The relation of the great sympathetic nerve to the spinal marrow does not appear to be the same in different species of animals, and at different periods of life. Before birth, it does not exist; but after that, it becomes daily more intimate and necessary to life, in proportion to the more perfect development of the functions dependent on that relation. In brute animals, this relation exists in a much inferior degree. We may easily conceive, then, that the sudden destruction of the spinal marrow in animals, whose functions are developed, must have a great degree of influence on the system of ganglions, of which it destroys the numerous sympathies; without being obliged to admit that it thence receives its origin.

It appears that the apparent opposition of the experiments of Legallois to the facts here related, may thus be readily explained; and that we may admit, notwithstanding those experiments, that the system of ganglions possesses in itself all the powers necessary for fulfilling its functions; that it has no extrinsic origin, but that the branches of communication which unite it to the spinal marrow establishes between them an intimate connexion.

M. Lallemand next considers the cause of the motions of the fœtus, which were noticed two days before its birth. He refers to the opinion of Reil and Prochaska, (an opinion which has been claimed both by Scarpa and Legallois,) that the smallest nervous filament possesses in itself the power of producing the nervous agent. Without pretending to defend this opinion, M. Lallemand remarks, that the nerves in the present case were as large and firm as those of an ordinary fœtus at the same age.

Whether such an opinion be admitted or not, it is equally necessary to investigate the cause of the muscular contractions which were observed. On speaking of the motions of the fœtus in the uterus, Bichât, from the probable entire absence of the functions of all the organs of *animal* life, attributed the cause of them to those of *organic* life. He thought that those, being alone in action, could alone transmit to the brain sensations capable of producing muscular contraction. He compared these motions to those of a person asleep, suffering from painful digestion. The brain not existing in the present instance, the influence of the system of ganglions on the nerves of animal life could only be excited by the numerous anastomoses of those ganglions with the cervical, brachial, lumbar, and sacral plexuses.

For the distinct characteristics of the nervous system of *organic life*, its distribution, and functions, we refer our readers to the work of Bichât.

We shall now consider the reflections of M. Lallemand on the *nervous system of animal life.*

Let us examine, he observes, if pathology will not furnish us with facts respecting the cerebrum, cerebellum, and spinal marrow, equally determined with those of the great sympathetic nerve, and with indications more applicable to man than experiments on brute animals.

Four years since, he saw at the Hôtel-Dieu, an acephalous fœtus, born at the full time, or nearly so, which lived three days. During the whole of that time it cried strongly, and performed the motions of sucking whenever it felt any thing between its lips: it was fed with milk and water sweetened with sugar,—for no nurse could be found who would suckle it. The motions of the limbs were free, and, when any thing was placed in the hands, the fingers were bent to grasp it; but the motions were less forcible than those of an ordinary fœtus. The cerebrum and cerebellum were entirely wanting; there only remained, at the base of the cranium, the spinal marrow a little elongated, and the annular protuberance, with the origins of the pneumo-gastric, trifacial, and optic nerves. A similar fact was lately observed at the Hospice de Perfectionnement. They are common in authors, and so well understood at the present day, that there is no necessity to adduce examples of it, as was done respecting the more rare cases of destruction of the spinal marrow.

These facts are sufficient to prove, that the brain is neither the *only source of nervous influence*, as Haller believed, nor the *sole centre of the nervous system of animal life*, as was the opinion of Bichât. They prove also, were it necessary to do so, that the motions independent of the will are not under the influence of the cerebellum.

There results from this, as an immediate consequence, that the organs which receive their nerves from the medulla oblongata and spinal marrow, derive thence the nervous influence which animates them; while it is from the cerebrum that arise the determinations of the will.

A greater number of acephalous fœtuses have been witnessed, in which respiration could not be established: in those instances, dissection has shown that the medulla oblongata had been destroyed with the rest of the brain. It is on the medulla oblongata, then, that the phenomena of respiration depend; and it is from that organ that the pneumo-gastric nerves take their origin.*

A question then arises,—if the medulla oblongata and spinal marrow furnish to the nerves which arise from them the influence

* See the work of Legallois on the Principle of Life, p. 248.

necessary for the performance of their functions, how is it that compression, or inflammation of the cerebellum, however small in extent, will cause in one case, palsy; in another, convulsions in the muscles which receive their nerves from the spinal marrow? This may be explained by what was before advanced respecting the disturbance caused from sudden injury to one set of functions, to those functions which sympathize with them. When the injury is more gradually produced, as Legallois observed on the destruction of the spinal marrow, such consequences do not arise. Thus we find that large tumors exist, which compress, and even considerably alter, the shape of the cerebellum; and suppuration of considerable portions of the cerebrum,—without being productive of paralysis, convulsions, or any of those consequences which ensue from sudden injury. These alterations of structure, coming on more or less slowly, accompanied with different symptoms according to the period of their existence, conducts us, by almost insensible gradations, from the acute diseases of which we have spoken, to the destruction of the cerebrum and cerebellum in the acephalous fœtus.

The following conclusions, it appears, may be deduced from the above-mentioned facts:—

1. All the nerves of animal life derive from the part from which they originate, whether the brain or spinal marrow, the nervous influence necessary to fulfil their functions.

2. It is from the cerebrum that arise the determinations of the will.

3. The cerebrum exerts on the spinal marrow an influence which is not confined to the direction of its action according to the will, but there also results from it an increase of energy in the functions of the spinal marrow.

4. The influence of the cerebrum is not the same on all parts of the spinal marrow; on those, for instance, which furnish the nerves for respiration.

5. This influence is greater, and more necessary, in proportion to the length of the period from the birth of the fœtus.

The different effects produced on different animals by galvanism, and the greater or less destruction of the brain, with the variation in the appearance of the spinal marrow and the size of the ganglions of the sympathetic nerve, give rise to some further reflections. In proportion as we diverge from man, in the animal creation, we find a diminution in the volume of the brain, and the number and depth of its convolutions; the cerebellum rests more behind the cerebrum, loses its lateral portions, and is, in fine, reduced to its central part. The spinal marrow, in man, forms a regular continuation of it, showing some slight protuberances only, about the origin of the nerves; but, as we descend in our observations, we find the ganglions of the spinal marrow become more and more distinct, and the cords of communication less thick. It appears that the functions of the different parts of the nervous system are proportionably more independent one on the other, as those parts are less perfectly developed, and their union less intimate. What proves it is, that in vertebral animals, which have a cerebral tuberosity, reproductions of certain parts may take place, but these reproductions have limits. In radiated animals, each distinct portion will produce an entire animal, provided care be taken to preserve the central ganglion; in annulated animals, each ring having a ganglion similar to all the others, can produce an entire individual:—we must remark, that these entire reproductions only take place in the last class of vertebral animals. If these anatomical differences can explain the cause of the differences observed in the results of experiments on animals, it must then be admitted that there is too great a difference between the brain of man and that of a rabbit, for example, to permit those experiments to induce us to dispense with the study of man from himself.

We have not referred to the experiments and observations of Dr. Wilson Philip in the consideration of this subject, being desirous to confine ourselves to the route taken by M. Lallemand: but we must here observe, that M. Lallemand has been anticipated in several of his opinions by that very ingenious physiologist. Our readers, we presume, are so well acquainted with the work of Dr. Philip, as to render any particular remarks of ours quite useless.

---

The following very interesting case of *Nymphomania* occurred a short time since to the observation of Dr. Jauzion, of Damietta. We present it to our readers in the words of Dr. J., from the same motives that induced him not to treat "de obscœnis" in the vulgar tongue. It may also be beneficial to some of our younger readers to observe the facility with which medical men on the continent write in the Latin language. Some of the sentences in the following narration are expressed in a manner which, for ease and delicacy, is hardly inferior to that of the younger Pliny.

"Virgo circitèr quadraginta annis nata, adhùc etiam menstruis purgationibus non carens, et se amore infelici jam diù excrucians, paucis ab hinc annis hominum societatem et consortium accuratè fugebat; tristis erat et cogitabunda, temperamento bilioso sanguineoque prædita, papulis rubris, quæ ipsius vultum crebrò deturpabant obnoxia, et à decem circiter annis, affectionibus hystericis non raró torquebatur. Triginta annis elapsis, ideæ magis ac magis, fiunt melancholicæ, vitam solitariam agebat et tantum-modò in ædem sacram ibat. Parochiæ animarum curator, ætate provectus et qui bonum sibi paraverat nomen, erat hujus unica consociatio. Per hunc statum melancholicum, tertio Aprilis die febre tertianâ correpta est; morbus autèm post octo dies, solis naturæ viribus evanuit. Aliquantò post, in totâ corporis superficie pruritum experitur, maximè in vultu, pustulis liventibus à juventute asperso, pro quibus sanandis dulcamaram, extractum hujus, serum lactis, balnea tepida, lac vicissim adhibebat medici ordinarii consilio. Hæc dùm geruntur evanescit ciborum appetentia, magna fit animi, corporisque mutatio: fulgent oculi solitò

magis: huc usque tamen accurata et rectè dixerat. Diè maii vigesimo nono, pentecostes autèm festo, pastor suprà dictus, ad quem sub obscuro mane iverat virgo, delirii erotici symptomata, actibus indecoris, verbisque flagitiosis et turpibus manifestata, primus animadvertit. Illicò ad parentes ipsemet lugens eam reconduxit. Virgini datur mulier pro sociâ; hanc autem recusat, dicens *se semper fœminas odio habuisse.* Tempore meridiano, in cassùm ad prandium advocatâ virgine, frater hujus cubiculum adiit et sororem suam terræ procumbentem et in faciem, horriferis capillis, recumbentem conspexit. Absente medico assueto, me advocant propinqui.

"Quæ suprà sunt à consanguineis mihi enarrata fuerunt; quæ verò infrà propriis oculis per triduum egomet vidi. Cùm ad ægrotam accedissem, eam sedentem in cathedrâ aspexi, vultu subrubro, fulgentibusque oculis; pulsus est celer, non æquis intervallis movetur; dolet, tumetque leviter hypogastrium; eam de doloribus interrogo, ad quæsita obstinat è respondere non vult. Datur ei ex succo limonum potio, in rusticæ mulieris astantis caput potionem et poculum simul atque conjecit. Suavitate sermonis mei hujus animum sedare frustrà conor. Multùm satietatis afferunt hæc verba; ab hinc semi-horâ elapsâ magno ejulatu advocor, et quod notatione dignum, eam declamitantem tertiam strophem odes *à Priape* dictæ invenio. Coràm me in quemdam virum illius custodem repentè irruit, ei dicens, propris verbis, omni verecundiâ depositâ, *ad opus te provoco, appetentiæ meæ celeritèr obsequere, aut tibi vitam eripio, veneris enim cupiditate ardeo.* Tunc frequentior pulsus, magis rubet facies, fiunt subsultus, fulgent oculi; eundem virum suprà dictum verbis flagitiosis et turpibus sollicitat. Velociter se proripuit foràs vir provocatus. Largiter, sed non sine negotio, à chirurgo missus est sanguis ex unâ venarum brachii; serum lactis nitratum, emulsiones frigidas, remedia torporem inducentia, clysteria refrigerantia, balnea semi-frigida frustrà adhibere voluimus, pertinaciter omnia respuit virgo. Interea rediit sacerdos et hanc ad tranquillitatem mentis, totis viribus perducere enititur; è lecto surrexit ægra furiam simulans et se nudam exhibens, ad me, pastor, veni, exclamat ea, horriferâ voce; sacerdotes enim imprimis amore prosecuta sum. Tunc ligantur pedes et manus, intereaque ad exorcismum recurrit pastor; mox in soporem incedit virgo, hæc verba voluptuarie proferens, *Hem! urinam expello,* et revera pudendæ partes humore quodam aspersæ fuerunt atrum odorem exhalante. Hanc animi tranquillitatem quæ repentè agitationi spasmodicæ successit exorcismo, bonâ fide, tribuerunt pastor atque astantes. Pulsus fit minùs frequens, subpallida facies et abundanter exsudans. Hypogastrium, antea tensum, minùs tumet, stupida visa est ægrotans. Interim accersivit Doctor Seganville vaurensis ægræ medicus ordinarius. Communi consensu duodecim hirudines partibus pudendis admotæ sunt, et horâ nonâ vespertinâ, in balneum semi-frigidum, per duas horas vi demissa est. Totâ nocte sat quieta fuit, sed perpetuò mussitabat; pulsus tunc debilis, respirandi difficultas; manum frequentèr in vaginâ admovebat, clitoris tumebat, sive hæc erectio tribuatur ipsimet naturæ morbi, sive pendeat à fricatione quâ utebatur ægrotans ex Astrucii verbis. Per hanc intermissionem corticem peruvianum largâ dosi in vanùm dare voluimus. Postero die circà horam decimam matutinam effrenata et furiosa veneris cupiditas repènte orta est, simùl è atque lecto surgit, subuculam deponit, scalas furentèr descendit et in lignarii sinum irruit, ei dicens *ad opus te sollicito*, *tàm formosam mulierem nusquàm possidebis.* In oris atque oculis medicorum et circunstantium tàm indecora provocatio facta fuit. In lecto per vim tunc collocata est, iterùmque ligantur pedes et manus, et invitis vinculis detenta fuit auxiliantibus quatuor fæminis corporis firmitate præditis. Hæc dùm geruntur, ad ægram revertit pastor, demones sacris incantationibus rursùs, sed frustrà fugat. Per septem circitèr horas, verba impura, provocationes impudicæ ex ore continuo affluebant, aderant motus convulsivi in vultu, pulsus frequens, nervosus, et omnia ferè alia symptomata in paroxismo precedente descripta; strangulatione spasmodicâ adstrictus erat æsophagi ductus, inde respirandi difficultas, et hac invitâ, quandoque verborum copia; astante pastore, astantibusque nobis medicis atque propinquis duo prima carmina primæ strophes odes suprâ dictæ declamitare incepit. Novem horis perseveravit hic paroxismus, desiitque ut antèa egestu, sive per vaginam, sive per urethræ canalem, humoris similis humori versùs finem antecedentis profuso. Sopor statim atque subsecutus est, pulsus fit debilitate notabilis. His symptomatibus virium jactura, urinarum dejectio copiosissima, singultus frequentes, risus sardonicus rapidè successerunt; sudor gelidus toto corpore manabat; tandem è medio abiit misera virgo sex aut septem horis post paroxismi finem.

"Si à propinquis cadaveris sectionem faciendi nobis concessa fuisset facultas, in organis genitalibus, hujus nymphomaniæcausa forsàn patefacta esset. Hypogastrii enim dolor constans, tamet si paululùm acutus, nisi assertionis nostræ veritatem demonstret, saltèm rem verisimilem reddit. Ab omni tamen consideratione erga hæc abstinebo, deficiente notione ex cadaveris sectione excerptâ."

---

*An interesting Memorial on the Distinction of Aneurisms of the Heart into active and passive,* has been published by M. Rostan;* in which he attempts to decide the question—Whether the causes which have been assigned for *active* aneurism of that organ, or that with increased thickness of its parietes; and for *passive* aneurism, or that with diminished thickness of the same parts; and the signs which have been attributed to those different lesions, are founded on actual and invariable facts?

M. Corvisart states, that "active aneurism of the heart acknowledges for its cause the sanguine temperament, strength of body, youth, violent efforts, long-continued journeys on foot or horseback, excess of venereal intercourse, the use of high-seasoned

* Nouveau Journal de Médecine, tome i.

food, excess in the use of spirituous liquors, singing, hollowing, and violent passions of the mind." The symptoms are "redness of the face; violence in the motions of the heart,—its action sensible to the eye and hand; strength, hardness, and vibration of the pulse; throbbing of the carotid arteries, &c." He attributes to passive aneurism, causes and symptoms the reverse of the above. Some cases, which have occurred to the observation of M. Rostan, seem to invalidate this distinction of the causes and symptoms of this disease. We have, indeed, sufficient facts already on record, to show that obstruction to the free passage of blood through either the arteries or veins, will, by calling forth greater exertions of the muscular power of the heart, produce increase of bulk and force of that organ, in almost all states of the general system. The individuals, who were the subjects of the observations of M. Rostan, were all of advanced age, living under the influence of causes essentially debilitating,—as a poor and spare diet, habitual inaction, want of amusement, and absence from their social connexions. Six cases of active aneurism of the heart, occurring under those circumstances, are related by the author; of which we shall transcribe two of the most interesting.

Frances Dumay, 65 years of age, of a weak and deteriorated constitution, had suffered for six months from violent throbbing of the heart, which came on after the healing of some old ulcers. Her face was red and suffused with blood, the eye-lids tumefied, and the superior extremities covered with scorbutic spots. Respiration was oppressed; and, about three o'clock in the morning, sensations of danger of suffocation came on, which disappeared in the course of the day. During these paroxysms, she was obliged to sit upright, and the throbbing of the heart was much increased; but the pulse was regular, although feeble. The appetite was good.

She had been repeatedly bled and blistered, without experiencing any relief. Slight anasarca came on soon after this; and she died about ten days after her admission into the Infirmary.

*Dissection.*—The right lobe of the *lungs* was more dense than natural, and was united to the pleura by old and firm adhesions. The left, had formed adhesions at its anterior surface, and somewhat resembled the spleen in appearance. There was a small quantity of fluid in the left cavity of the chest.

The *heart* was of an enormous size, firm, and the parieties of the left ventricle were increased in thickness at least eighteen lines; its cavity was about the size of a large nut.

The aorta was ossified and rugous on its interior surface, and alternately dilated and contracted throughout the whole of its course through the cavity of the chest: it was not examined further.

The parts contained in the abdominal cavity were in a healthy state.

M. M. Lacour, aged 82, was admitted into the Infirmary in a state of general debility, attributed solely to her advanced age: the state of weakness, which had come on gradually, was such that she could not move her limbs. Her face was suffused with blood, and several violet-coloured patches were dispersed on it. Her intellectual faculties and organs of sense were in a state of almost perfect inaction; the digestive functions were not deranged, although not very active. Her respiration was rattling, particularly during sleep; and she sometimes coughed, but without expectoration. The pulse was small, soft, and irregular.

The debility increased; and an excoriation of the integuments of the sacrum took place, which became gangrenous, and produced febrile symptoms. She suffered much pain from this affection, and died about a fortnight after her admission.

*Dissection.*—The cavities of the thorax contained a considerable quantity of serum; the lungs were in a healthy state.

The parieties of the left ventricle of the *heart* were very thick and firm, and its cavity almost obliterated. There were portions of ossification, of about the size of a filbert, around the aortic and aurico-ventricular orifices.

The ventricles of the *brain* were filled with serum; which was also found in considerable quantity between the tunica arachnoides and pia mater.

The *cerebellum*, towards its posterior part, was of a remarkably firm consistence, analogous to the tissue of the trunks of the large nerves.

The other cases universally presented ossification of the coats of the aorta, or about its orifice; and a preternaturally firm, dense structure of the lungs,—or, as M. Rostan expresses it, they were *splenified*. The patients were all in a state of great debility; and the pulse was small, weak, and generally irregular.

"The above facts," the author observes, "prove that active aneurism of the heart is very frequent in old people; and that it, in the greater number of instances, arises from ossification of the aorta. Whence it appears that youth, adult age, the sanguine temperament, and all the train of general exciting causes, are not always necessary for the production of active aneurism; that the pulse, far from being always *strong, hard*, and *vibrating*, is often *small*, *contracted, soft*, and *hardly sensible*; that these characters are strongly marked as the obstacle to the circulation is greater, and the heart has thence necessarily acquired greater bulk and power; and that the distinction of aneurisms into *active* and *passive* is not admissible in these cases, neither with relation to their causes nor their symptoms."

The author then proceeds to make some reflections on these diseases. "Aneurism of the heart," he observes, "is of such frequent occurrence in old people, that few die at an advanced age without being more or less affected by that disease. The fear of the imputation that we see this disease universally, has not prevented our making this assertion, since it is the result of facts observed in the examination of bodies, which the immense population of the Saltpetriere Hospital, with the great age of the persons therein admitted, give us frequent opportunities of confirming.

"Corvisart attributes the formation of aneurism of the heart 'to obstacles offered to the course of the blood, either from faulty organization, or some morbid state, the consequence of the influence of moral affections on the function of the heart; perhaps, also, from the greater or lesser stimulating quality of the blood, which, in an equal quantity, might augment or diminish the action of that organ.' Experience has shewn that an accumulation of calcareous phosphate in the large vessels, an inevitable result of the progress of age, has been in many instances the cause of aneurism. This accumulation of phosphate of lime, by diminishing the elasticity and calibre of the vessel, excites the increased action of the heart to overcome this unnatural resistance, and thence an increase of its muscular structure. Four years since, on opening a body, we met with a heart of a remarkably large size, without witnessing the state of ossification usual in such cases. The subject was abandoned with an observation, that obstruction to the course of the blood probably existed at some distance from the heart; when some students who were present, desirous of ascertaining the fact, pursued the examination of the aorta, which they found ossified, and almost obliterated, from its entrance into the abdomen to its division into the iliacs. We observed the same thing a few days since.

"We have noticed in persons who have the bones of the chest much distorted, and whose lungs are confined within a narrow space, that the parieties of the *right* ventricle of the heart have been much thickened, whatever might have been the age and constitution of the patient. The obstacle being in the pulmonary circulation, the development of the force to overcome it, is consequently in the organs of this circulation. We have lately witnessed three examples of this fact. Bayle has remarked that, in several phthysical patients with oppressed respiration, he has found the right ventricle of the heart augmented in thickness and volume; which can only be attributed to the obstruction to the circulation through the lungs, which the tubercles have afforded. M. Jadelot has noticed the same thing in children, in whom the tubercles have compressed the pulmonary artery.

"In the greater number of instances, the obstacle which has obstructed the free course of the blood may be found, if it be sought with assiduity, either about the orifice of the heart, or at a short distance from it. Might not ossification of the smaller arteries produce the same effect?"

Senac, in his Treatise on the Diseases of the Heart, has noticed all the different alterations from the state of health, of which this organ is susceptible; but he concludes the greater number of his chapters with saying, that these alterations furnish, during life, no signs by which their existence may be discriminated; and that, although we might be able to recognize them, our art probably will not furnish us with the means for their relief.

